# Hypophosphatemia and FGF23 tumor-induced osteomalacia in two cases of metastatic breast cancer 

**DOI:** 10.5414/CN110242

**Published:** 2020-11-16

**Authors:** Matthew Abramson, Ilya G. Glezerman, Maya Srinivasan, Richard Ross, Carlos Flombaum, Victoria Gutgarts

**Affiliations:** 1 Renal Service, Memorial Sloan Kettering Cancer Center,; 2 Weill Cornell Medical College,; 3 SUNY Downstate, and; 4 Department of Medicine, Memorial Sloan Kettering Cancer Center, New York, NY, USA

**Keywords:** fibroblast growth factor 23, tumor-induced osteomalacia, osteogenic osteomalacia, hypophosphatemia, klotho, metastatic breast cancer

## Abstract

Tumor-induced osteomalacia (TIO) is a rare paraneoplastic syndrome characterized by factor-induced dysregulation of phosphate and vitamin D metabolism resulting in alterations in bone formation, leading to bone pain and fractures. While the true incidence is likely underestimated, less than 500 cases of TIO have been reported since initial description in 1947. TIO cases have classically been associated with mesenchymal tumors of bone and soft tissue, but have also rarely been linked to malignant tumors, with scant reports implicating non-mesenchymal tumors. TIO is mediated through inappropriate tumor overproduction of fibroblast growth factor 23 (FGF23). Increased FGF23 secretion leads to hypophosphatemia by (1) reduced phosphate reabsorption via activation of the proximal renal tubular epithelial cells to internalize sodium phosphate cotransporters and (2) reduced activation of vitamin D3 via inhibition of the renal enzyme 1-α hydroxylase. Low circulating levels of active vitamin D lead to reduced intestinal phosphate absorption and impaired mineralization of osteoid matrix. TIO in breast cancer poses a distinct diagnostic challenge due to the common adjunct oncologic management with bone protection therapy such as denosumab or bisphosphonates. These agents can be culprits of hypophosphatemia and hypocalcemia, rendering timely diagnosis of TIO difficult. Delay of diagnosis of TIO can result in worsening functional status, and early morbidity and mortality. To date, there has been one prior case report of TIO in breast cancer, and herein we describe two additional cases of TIO in this setting.

## Introduction 

Fibroblast growth factor-23 (FGF23) is a phosphaturic humoral factor produced by osteoblasts and osteocytes [1]. First identified two decades ago, mutations in the cleavage of FGF23 cause several inherited renal phosphate wasting diseases leading to rickets in children or osteomalacia in adults [2, 3]. In the paraneoplastic setting, FGF23 oversecretion leads to tumor-induced rickets/osteomalacia (TIO) also known as oncogenic osteomalacia [4]. TIO is typically reported with mesenchymal tumors [5, 6], and is starting to become recognized in patients with liquid [7] and solid organ malignancies [8, 9, 10] as well. 

FGF23 is a key regulator of phosphate metabolism. The primary physiologic function is to lower serum phosphate levels which is mediated by FGF receptors (FGFR) and klotho complexes [3]. FGF23 downregulates the expression of cotransporters in the kidney that are essential for the reabsorption of phosphate. Additionally, FGF23 downregulates the expression of enzymes that activate vitamin D which increases intestinal phosphate absorption, thereby indirectly lowering serum phosphate levels [11]. 

Phosphate is primarily found in bone and is responsible for skeletal strength and rigidity. Low phosphate levels manifest as general muscle weakness, fatigue, and in extreme cases impaired cardiac and respiratory function [12]. These symptoms, in patients with cancer, may be attributed to their malignancy, and the potential diagnosis of TIO may be overlooked, especially with the rarer non-mesenchymal origin tumors. Below are examples of two case reports of patients with metastatic breast cancer with severe hypophosphatemia, phosphaturia and elevated serum FGF23, consistent with TIO. To the best of our knowledge, there is only one other case report of TIO associated with metastatic breast cancer [13]. These cases are particularly challenging given the use of antiresorptive therapy in patients with bone metastasis which can trigger FGF23 overexpression [13] and worsen underlying oncologic osteomalacia. 

## Case 1 

**Figure 1:**
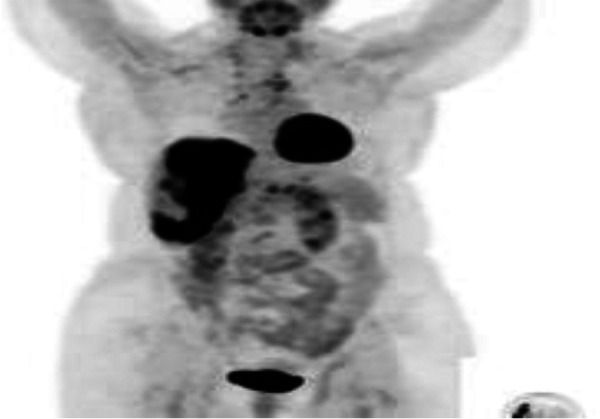
PET scan showing progression of disease for case 1. Metastasis to the liver, sternum, and sclerotic osseous lesions to the spine and right iliac.

A 47-year-old woman with metastatic breast cancer with liver and bone involvement was referred to the nephrology clinic for persistent hypophosphatemia. Seven years ago patient was diagnosed with left mammary duct carcinoma and underwent partial mastectomy followed by chemotherapy with paclitaxel and tamoxifen. She had a reoccurrence 3 years later and failed multiple lines of chemotherapy including eribulin and vinorelbine with last positron emission tomography (PET) scan showing metastasis to the liver, sternum, and sclerotic osseous lesions to the spine and right iliac (Figure 1). 

The patient was initiated on monthly denosumab for 1 year (12 doses in total) prior to the current nephrology visit, with last dose 1 month ago, to address metastatic bone involvement. Phosphorous level on consultation was < 0.9 mg/dL (2.5 – 4.5 mg/dL) with no prior levels. Remainder of bloodwork is shown in Table 1 which highlights low calcium 7.4 mg/dL (8.5 – 10.5 mg/dL) and elevated alkaline phosphatase (ALP) of 738 U/L (≤ 130 U/L). The fractional excretion of phosphate (FePhos) in the urine was elevated at 56% (< 5 – 10%). 

Etiology for hypophosphatemia was initially thought to be secondary hyperparathyroidism given elevated parathyroid hormone (PTH) of 488 pg/mL (12 – 88 pg/mL) due to hypocalcemia in the setting of recent denosumab administration. Phosphorous levels remained low despite oral calcium and phosphate repletion and oral calcitriol administration (Table 1). Given persistent hypophosphatemia, FGF23 was checked, and levels returned strikingly elevated at 2,430 RU/mL (≤ 180 RU/mL) suggesting an FGF23 secreting tumor as the most likely cause for severe hypophosphatemia. Unfortunately, the patient passed away within 1 month due to disease progression. 

## Case 2 

**Figure 2:**
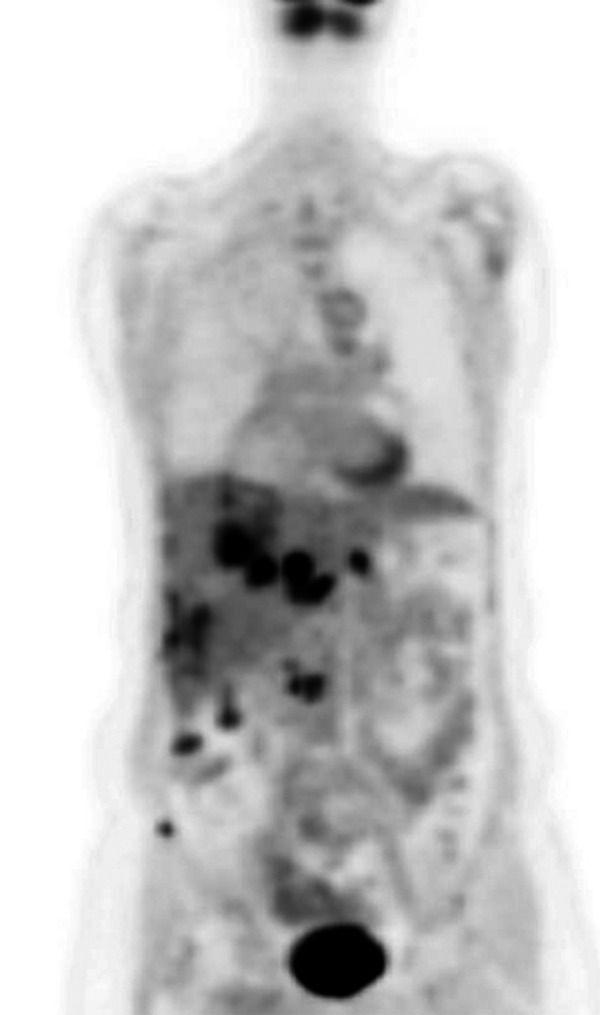
PET Scan showing progression of disease for case 2. Metastasis to the liver, right acetabulum, thoracic vertebrae, and right ilium.

A 55-year-old woman with triple negative invasive ductal breast cancer, who achieved remission 10 years ago presented with progressive weakness. She was found to have relapsed disease involving the liver, lung, and bone (vertebral, acetabulum, and ilium) 1 year ago (Figure 2), and subsequently received chemotherapy including palbociclib, nivolumab, and abraxane as well as 4 monthly doses of zoledronate, followed by 10 monthly treatments of denosumab. She last received bone-stimulating therapy and chemotherapy 3 months prior to admission. She had no other comorbidities, nor a history of additional medications or herbal supplements. She was a lifetime nonsmoker. She was admitted for obstructive jaundice due to progression of disease. During the course of her admission, she complained of severe lower extremity bone pain limiting ambulation. Prior to admission, the patient’s electrolytes were within normal limits. 

Upon admission, she was cachectic (body mass index < 18), with hypophosphatemia of 1.6 mmol/L (2.5 – 4.5 mmol/L). Nephrology was called for further evaluation. Remainder of lab studies are shown in Table 2 and include a normal corrected calcium of 9.5 mmol/L (8.5 – 10.5 mmol/L), low 25-hydroxyvitamin D of 15 ng/dL (20 – 50 ng/dL), elevated PTH of 287.3 pg/mL (12 – 88 pg/mL), and elevated ALP of 635 U/L (≤ 130U/L). FePhos was 78% (< 5 – 10%), consistent with phosphate wasting. Of note, 1,25-dihydroxyvitamin D was elevated at 83 pg/mL (20 – 50 pg/mL) despite not being on calcitriol. 

Given elevated urine phosphate, an oncologic osteomalacia was suspected and FGF23 was checked and was elevated at 548 (< 180) RU/mL. Due to aggressive supplementation, serum phosphate increased to a peak value of 3.8 mmol/L; PTH decreased to 44, but FGF23 and FePhos remained elevated at 424 and 72%, respectively. The patient continued to decline and passed away within 2 weeks. 

## Discussion 

FGF23 is a glycoprotein part of the FGF family which is subdivided into 7 subfamilies with 22 members reported in humans [14]. FGF23 belongs to the FGF19 subfamily which has also been called the endocrine FGFs due to the inner protein structure allowing it to function as a circulating hormone [15]. FGF23 is derived from bones, and under physiologic conditions, its production is stimulated by extracellular phosphate. Once secreted from osteoblasts and osteocytes, FGF23 plays a pleiotropic role which links the bone with several organ systems including the kidney, heart, and cells part of the immune system [1]. FGF23 signaling contributes to regulation in cellular proliferation, survival, and differentiation making it an attractive pathway to hijack by cancer cells [16]. 

### FGF23 renal pathophysiology 

**Figure 3:**
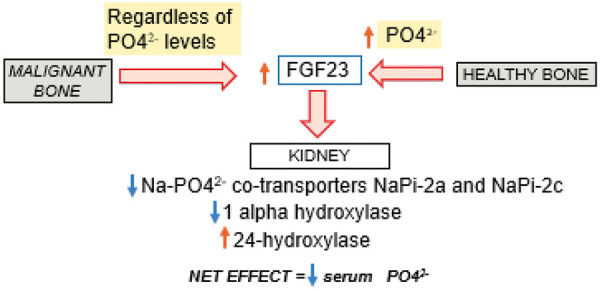
Bone-kidney axis and phosphaturic effects of FGF23. FGF23 is produced in bone by osteocytes in response to high serum phosphorous. In malignant bone, FGF23 is produced regardless of serum phosphorous. One of FGF23 targets is the kidney. FGF23 binds to FGR receptors and complexes with klotho on the basolateral surface of proximal tubular cells. This causes a decrease in expression of sodium-phosphorus co-transporters (Na-PO4^2-^) whose role is renal phosphate reabsorption. Indirect effects include inhibition of 1-α-hydroxylase levels which are necessary to activate vitamin D and increased expression of 24-hydroxylase which degrades active vitamin D. The net effect is a decrease in serum phosphorous.

With respect to the kidney, the main function of FGF23 is to lower serum phosphate levels as shown in Figure 3. This is established through direct inhibition of phosphate reabsorption at the level of the proximal tubular cells, and indirectly by downregulation of enzymes necessary to activate vitamin D. Direct actions involve the binding of circulating FGF23 to FGF receptors (FGFRs) and coreceptor klotho on the basolateral surface of the proximal tubular cells. This results in decreased expression of two sodium-phosphate cotransporters called NaPi-2a and NaPi-2c. These transporters, located on the apical surface of the proximal tubular cell are responsible for renal phosphate reabsorption. Decreased expression of NaPi-2a and NaPi-2c is therefore a direct cause of phosphaturia [17]. 

FGF23 also indirectly lowers serum phosphate levels by inhibiting renal 1-α-hydroxylase which is necessary to activate vitamin D. Further, FGF23 also increases the expression of 24-hydroxylase which degrades the active form of vitamin D into inactive metabolites. These actions collectively reduce active levels of vitamin D leading to decreased intestinal reabsorption of phosphate [18]. This relationship has been demonstrated in animal studies where a single injection of recombinant FGF23 resulted in reduction of serum phosphate and 1,25 (OH) 2D levels independent of PTH levels [11]. During the experiment, PTH levels remained low, and the hypophosphatemia was reproduced by injection of FGF23 in parathyroidectomized rats [11]. 

### FGF23 mode of inheritance 

Both genetic and acquired mechanisms of FGF23-related hypophosphatemic disease have been described. Genetic mechanisms vary by mode of inheritance. Autosomal dominant hypophosphatemic rickets (ADHR) is caused by mutations in *FGF23* gene [2]. The autosomal recessive variant is caused by mutations in dentrin matrix protein 1 (DMP1) [19]. The X-linked dominant form occurs due to mutations in phosphate-regulating gene (*PHEX*) [20]. 

An acquired FGF23 hypophosphatemic disease is associated with the administration of intravenous iron, specifically the saccharated ferric oxide and iron polymaltose. Evaluation of these patients showed elevated FGF23 levels with the exact mechanism not known [21]. TIO is another example of an acquired form of FGF23 hypophosphatemic disease [17] which is reviewed in greater detail below. 

### Tumor-induced osteomalacia 

TIO is a rare paraneoplastic disease, first described in 1947 by Robert McCance who reported a patient with pain and weakness in the setting of low phosphate levels. His symptoms persisted despite being treated with vitamin D, and eventually improved only after a tumor found in the femur bone was resected [22]. Animal experiments have supported the presence of the humoral factor leading to hypophosphatemia [23]. The earliest evidence to support this in humans was done by Miyauchi et al. [24] where tumor removal in a patient with osteomalacia and injection into healthy mice lead to hypophosphatemia. 

Tumors associated with TIO are usually mesenchymal in origin [17]. Within the reported cases of TIO, 40% occur in the bone and 55% occur in soft tissues. The thigh and femur are the most common sites of involvement with the pelvis reported in only 8% of cases, and only 2% of cases reported as involving more than one site [25]. These tumors can be histologically polymorphous, but in 1991 Weidner [26] proposed a classification system to divide them into four morphologic patterns including phosphaturic mesenchymal tumor mixed connective tissue variant (PMTMCT), osteoblastoma-like variant, non-ossifying fibroma-like variant, and ossifying fibroma-like variant. PMTMCT comprises 70 – 80% of cases of TIO and typically begins in bone or soft tissues [5]. 

Non-mesenchymal tumors with TIO manifestations are now being recognized and reported in leukemia [7], B cell non-Hodgkin’s lymphoma [8], sarcoma [10], and other solid organ cancers including lung [27], prostate [28], and colon cancer [29]. There is only one case of TIO reported in metastatic breast cancer [13] with the two cases above resulting in a total of three. During malignancy, abnormal FGF signaling has been shown to induce cell proliferation and angiogenesis thereby promoting metastasis [16]. In breast cancer specifically, molecular alternations in FGFR1 and FGFR2 receptors are the most common reported [16]. Clinical trials support this data where phase I trials showed hyperphosphatemia as the most common adverse effect when novel tyrosine kinase inhibitors targeted FGF signaling [30]. 

Diagnostic evaluation of TIO should start with a comprehensive metabolic panel to check serum phosphorous and calcium levels which are typically low. Alkaline phosphatase may be elevated as in case 1 (738 U/L) and case 2 (635 U/L) due to osteoblast hyperactivity. Vitamin D levels should be checked and are typically low due to the inhibitory effect of FGF23. This was seen in our cases where vitamin D levels were 8 ng/mL and 15 ng/mL in case 1 and 2, respectively. PTH levels may be variable and increased at times as part of a normal feedback response to low vitamin D levels and subsequently hypocalcemia. In both cases, the elevation in PTH (488 pg/mL and 287 pg/mL) was likely multifactorial; initially as a feedback to hypocalcemia in the setting of denosumab. Secondary hyperparathyroidism has been demonstrated in patients receiving denosumab as a result of prolonged hypocalcemia caused by this drug [31], leading to renal phosphate wasting in some patients. This mechanism may have contributed to pathogenesis of hypophosphatemia in our patients. However in case 1, phosphorus remained low despite aggressive supplementation. Persistent hypophosphatemia however should also raise concern for an FGF23 secreting tumor. For case 2, denosumab was given 3 months prior to recognition of hypophosphatemia. Furthermore, FGF23 remained elevated, and phosphaturia continued despite PTH normalization. Therefore, denosumab likely did not play a major role in the FGF23 elevation or renal phosphate wasting. Along with serum FGF23, urine studies including urine creatinine and urine phosphorous must be checked to calculate the fractional excretion of phosphate and tubular reabsorption of phosphate. In the setting of TIO, one would expect a high fractional excretion of phosphate (> 10%) and low tubular reabsorption of phosphate (< 75%) due to inhibition of sodium phosphate transporters at the proximal tubules and low vitamin D. Dihydroxyvitamin D-1,25 was low in case 1 as expected due to suppressed activation by FGF23. However, in case 2, dihydroxyvitamin D-1,25 was elevated in the absence of calcitriol. Although in patients with chronic kidney disease and hyperphosphatemia FGF23 is elevated leading to suppression of vitamin D 1,25 production, we hypothesize that perhaps in some patients with hypophosphatemia, other mechanisms may be responsible for higher vitamin D 1,25 levels to counteract effects of low phosphorus levels. 

Several imaging modalities can be used to identify the tumor, including magnetic resonance imaging (MRI) and PET scan. Somatostatin receptors (SSTR) based functioning imaging can also be performed since some of these tumors express SSTRs [32]. However, clinicians have to be mindful that inflammatory reactions can cause a false positive SSTR imaging [32]. In cases where tumor is identified, the treatment of choice is resection. Once FGF23 levels decline in circulation, serum phosphate levels return to normal, as early as five days post operatively [33]. In cases where the tumor is inoperable, medical management may be attempted with phosphate supplementation and calcitriol as recommended in our cases of metastatic disease. Octreotide is another potential treatment, given link with SSTR. Targeted antibodies against FGF23 have shown promise in animal models [34]. 

## Conclusion 

TIO can be a challenging diagnosis to make, especially in patients with malignancy other than mesenchymal origin, as symptoms of hypophosphatemia are nonspecific and could be easily attributed to the underlying cancer. In fact, the average time from recognition of osteomalacia to identifying the associated tumor is ~ 5 years [35]. We recommend more frequent testing of serum phosphorous since it is not part of the routine basic metabolic panel. Furthermore, in breast cancer specifically, patients are frequently managed with bone-targeted therapy such as bisphosphonates and denosumab which can further exacerbate hypophosphatemia. Antiresorptive therapy during malignancies should be carefully weighed with degree of hypophosphatemia and risk of skeletal-related events. Patients with TIO should be evaluated for resection, which can be curative when involving a solitary lesion. It is reasonable to check FGF23 levels in oncologic patients with persistent hypophosphatemia despite adequate supplementation of phosphorus and vitamin D and discontinuation of the drugs known to cause renal phosphate wasting. In patients with several lesions or metastatic cancer such as described above, systemic oncologic therapy and supplementation of phosphorous, calcium, and vitamin D can be attempted to improve the quality of life. 

## Funding 

This research was supported by National Institute of Health grant award P30CA008748. 

## Conflict of interest 

Ilya Glezerman owns Pfizer Stock. Remaining authors have nothing to disclose. 

**Table 1.** Case 1. Sequence of laboratory findings and treatment for hypophosphatemia.

	–12 months to –1 month	–10 days	–4 days	Nephrology consult (day 0)	+10 days
Treatment					
Denosumab (mg)	120 mg/monthly × 10 doses				
Potassium-phosphate/sodium-phosphate (mg)				250-45-298 t.i.d.	250-45-298 t.i.d.
Calcitriol (mcg)				0.25 b.i.d.	0.25 b.i.d.
Laboratory studies					
Serum phosphate (mg/dL)			< 0.9	1	1.1
Serum calcium* (mg/dL)	Range 8.7 – 10.5	8.1	8.4	7.9	9.1
Alkaline phosphatase (U/L)	Range 97 – 506	504	690	738	619
Serum PTH (pg/mL)				488	
Serum FGF23 (RU/mL)				2,430	
Serum 25-OH Vit D (ng/mL)				8	
Urine sodium (mEq/L)				22	
Urine calcium (mg/dL)				< 1	
Urine phosphate (mg/dL)				214	
Urine creatinine (mg/dL)				229	
FePhos**				56%	

*Corrected calcium = total calcium (mg/dL) + 0.8 (4.0-serum albumin [g/dL]), where 4.0 represents the average albumin level. **FePhos = (urine phosphorus/serum phosphorus) × (serum creatinine/urine creatinine). PTH = parathyroid hormone; FGF23 = fibroblast growth factor 23; FePhos = fractional excretion of phosphorus.

**Table 2.** Case 2. Sequence of laboratory findings and treatment for hypophosphatemia,

	–12 months to –3 months	–10 days	–4 days	Nephrology consult (day 0)	+3 days	+4 days
Treatment						
Denosumab (mg)	120 mg/monthly × 10 doses					
Potassium-phosphate/Sodium-phosphate (mg)				250-45-298 once	250-45-298 TID	250-45-298 QID
IV Phosphate (mmol)		30	30			15
PO calcium citrate (g)						3.8
IV calcium gluconate (g)				4		2
Laboratory studies						
Serum phosphate (mg/dL)	2.6 (month –3)	1.6	1.4	1.4	3.8	2.4
*Serum calcium (mg/dL)	9.2 – 10.1 range	9.0	8.7	8.0	9.4	9.3
Alkaline phosphatase (U/L)	138 – 253 range	516	581	712	677	664
Serum PTH (pg/mL)				287.3	44.3	
Serum FGF23 (RU/mL)				548	424	
Serum 25-OH Vit D (ng/mL)				15		
Serum 1,25-Dihydroxyvitamin D (pg/mL)				82		
Urine sodium (mEq/L)				< 20		
Urine calcium (mg/dL)				3.1		
Urine phosphate (mg/dL)				175	416	
Urine creatinine (mg/dL)				80	99	
**FePhos				78%	72%	

*Corrected calcium = total calcium (mg/dL) + 0.8 (4.0-serum albumin [g/dL]), where 4.0 represents the average albumin level. **FePhos = (urine phosphorus/serum phosphorus) × (serum creatinine/urine creatinine). PTH = parathyroid hormone; FGF23 = fibroblast growth factor 23; FePhos = fractional excretion of phosphorus.
